# Impaired Prosodic Processing but Not Hearing Function Is Associated with an Age-Related Reduction in AI Speech Recognition

**DOI:** 10.3390/audiolres15010014

**Published:** 2025-02-08

**Authors:** Björn Herrmann, Mo Eric Cui

**Affiliations:** 1 Rotman Research Institute, Baycrest Academy for Research and Education, 3560 Bathurst St., North York, ON M6A 2E1, Canada; mcui@research.baycrest.org; 2 Department of Psychology, University of Toronto, Toronto, ON M5S 1A1, Canada

**Keywords:** aging, text-to-speech synthesis, artificial intelligence, prosody, hearing loss

## Abstract

Background/Objectives: Voice artificial intelligence (AI) technology is becoming increasingly common. Recent work indicates that middle-aged to older adults are less able to identify modern AI speech compared to younger adults, but the underlying causes are unclear. Methods: The current study with younger and middle-aged to older adults investigated factors that could explain the age-related reduction in AI speech identification. Experiment 1 investigated whether high-frequency information in speech—to which middle-aged to older adults often have less access due sensitivity loss at high frequencies—contributes to age-group differences. Experiment 2 investigated whether an age-related reduction in the ability to process prosodic information in speech predicts the reduction in AI speech identification. Results: Results for Experiment 1 show that middle-aged to older adults are less able to identify AI speech for both full-bandwidth speech and speech for which information above 4 kHz is removed, making the contribution of high-frequency hearing loss unlikely. Experiment 2 shows that the ability to identify AI speech is greater in individuals who also show a greater ability to identify emotions from prosodic speech information, after accounting for hearing function and self-rated experience with voice-AI systems. Conclusions: The current results suggest that the ability to identify AI speech is related to the accurate processing of prosodic information.

## 1. Introduction

The advent of modern artificial intelligence (AI) provides a host of opportunities and innovations in technology and consumer markets [1,2,3]. One advancement, involving increasingly wide-spread adoption, is the synthesis of modern AI-based speech, through which text is converted to spoken speech with high naturalness [4,5]. For example, Google, Amazon, Meta, Microsoft, and OpenAI are some of the leading companies that offer advanced AI-based text-to-speech synthesizers to the public. Because it requires little to no computer programming skills to generate highly natural, synthesized speech, encountering voice-AI systems is increasingly common in everyday life, for example, in automated phone services, social chat bots, and voice assistive systems (e.g., Siri, GPS navigation support, etc.). Understanding how individuals perceive and experience modern AI-based synthesized speech is thus highly relevant to advance the technology with a user perspective in mind.

Most previous research on the perception of modern AI-based synthesized speech has focused on younger adults and the degree to which synthesized speech is intelligible and perceived as natural or human-like [6,7,8,9,10]. Work has further focused on the perceived valence of human vs. AI speech [7], the social attitudes towards AI speech with different accents [11], and the way younger adults communicate with voice-AI systems compared to humans [12,13,14,15]. However, little research has been concerned with how middle-aged to older adults perceive and experience modern AI-based speech, leaving the development of this critical technology age-uninformed.

One recent study that focused on younger and older adults demonstrated that AI speech can be used to investigate common speech-in-noise perception phenomena [16]. This research further showed that middle-aged to older adults, compared to younger adults, find AI speech more naturalistic and human-like, recognize less frequently when AI speech is presented to them unknowingly, and are less able to categorize whether the speech they heard was computer generated (AI speech) or spoken by a human [16]. Being less able to recognize computer-generated speech may put people at risk of scams or unwanted automated phone calls or at least leaves them potentially uninformed about the context of a communication situation. Although this previous research shows clear evidence for an age-related reduction in the ability to recognize AI speech, the underlying causes of the reduced ability are unclear [16].

**Figure 1:**
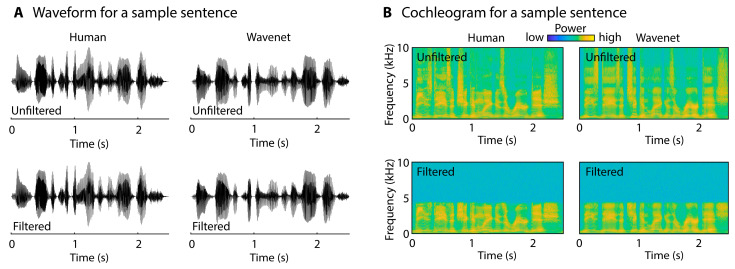
Sample stimulus representations. (**A**) Sample waveforms for the same sentence spoken by a human female speaker and synthesized using Google Wavenet separately for the unfiltered original speech and the 4 kHz low-pass filtered speech. (**B**) Cochleograms [76] of the sentences in panel (**A**).

Many adults aged 50 or older live with some form of hearing loss [17,18,19] that can make speech comprehension difficult, especially when speech is acoustically degraded or masked by background sound [20,21,22,23]. The causes underlying the difficulties with hearing and speech comprehension are multi-faceted [24,25,26], but middle-aged to older adults most commonly exhibit increased thresholds for the detection of pure tones at high sound frequencies—e.g., above 4 kHz [24,27,28]. High-frequency information in sounds and speech is thus often insufficiently accessible to middle-aged to older adults. Moreover, high-frequency information in AI speech appears less detailed compared to speech spoken by humans (see spectrograms in Figure 1; see also [16]), potentially providing an important cue for AI speech identification that may be less available to middle-aged to older adults due to hearing loss at high frequencies. Although previous work did not observe a correlation between hearing abilities and AI speech identification [16], a more direct examination may be warranted given the high prevalence of and substantial changes associated with age-related hearing loss [17,19,29]. Specifically, if information in the high-frequency range of speech enables younger but not middle-aged to older adults (due to high-frequency hearing loss) to recognize AI speech, then hearing loss could explain the reduced ability of middle-aged to older adults to identify AI speech. Removing high frequency information in speech should then reduce AI speech identification performance for younger adults and should equate performance between age groups.

Prosodic cues may be another relevant component that individuals use to identify AI speech. For example, acoustic analyses of AI speech revealed that the variations in prosodic properties across different speech segments can be reduced compared to the variations that are present in speech spoken by a human [16]. Older adults have been shown to experience difficulties in the processing of emotional messages conveyed through prosodic information in speech [30,31,32,33,34,35,36,37,38,39,40,41]. For example, older adults are less able to identify prosody-conveyed emotions—such as anger, sadness, and surprise—compared to younger adults [39,42]. The underlying causes are not fully understood but appear insufficiently explained by age-related hearing loss [42,43,44,45]; but see [30]. Nevertheless, a relationship between a reduced ability to identify AI speech and a reduced ability to recognize emotions through prosody, although not providing a mechanistic explanation, would deliver the critical insight that perhaps some of the inability of middle-aged to older adults to identify AI speech is related to prosodic processing.

The current study will assess the extent to which hearing loss and prosody processing contribute to the age-related reduction in identifying AI speech. In two experiments, younger and middle-aged to older adults listened to sentences spoken by different humans and sentences synthesized using different voices of Google’s AI-based text-to-speech synthesizer [5]. Participants were asked to categorize whether speech was spoken by a human or was computer-generated. In Experiment 1, we investigated whether high-frequency information in speech, to which middle-aged to older adults should be less sensitive than younger adults due to high-frequency hearing loss, is critical for the categorization of speech. To this end, participants categorized speech as human or computer generated for full-bandwidth speech or speech that was low-pass filtered at 4 kHz. We hypothesized that, if high-frequency information is important for recognizing AI speech, removing high-frequency information reduces AI speech identification performance for younger but not older adults. In Experiment 2, participants additionally performed a speech-emotion-judgement task to examine the extent to which a person’s ability to identify emotions from prosodic information in speech predicts how well a person is able to discriminate between human and AI speech. We hypothesize that a greater ability to identify emotions from prosodic information is associated with better AI speech recognition.

## 2. General Methods

### 2.1. Participants

Participants were recruited on Amazon Mechanical Turk (MTurk) using the Cloud Research interface [46]. Only individuals who indicated on MTurk/Cloud Research that they were born in the United States of America and that their native language is English were allowed to participate. Participants who self-reported having a history of a neurological disease or using a hearing aid were excluded. Demographic details about participants and the number of excluded participants are provided below for each experiment. Participants provided informed consent and received USD 7.5 (Experiments 1) or USD 11 (Experiments 2) at the same hourly rate. Participants were enabled to participate in only one of the two experiments. The study was conducted in accordance with the Declaration of Helsinki and the Canadian Tri-Council Policy Statement on Ethical Conduct for Research Involving Humans and was approved by the Research Ethics Board at the Baycrest Centre for Geriatric Care (REB #21-04; initial approval: 23 February 2021; annually renewed).

### 2.2. Experimental Setup

Experiments were conducted online in an internet browser. Experimental scripts were written in JavaScript/html, using jsPsych JavaScript libraries (Version 7.2.1 [47], stored at an online repository (https://gitlab.pavlovia.org, accessed on 1 January 2024), and hosted via Pavlovia (https://pavlovia.org/, accessed on 1 January 2024) to which participants were linked via MTurk. No specifications as to the type/brand of equipment participants should use (e.g., computer, screen, operating system, etc.) were provided, but participants were asked to use headphones.

### 2.3. Auditory Setup

At the beginning of the experiment, participants completed a volume check to set their computer volume to a comfortable level. Speech during the main task procedures was presented under clear conditions and perception of clear speech should have been unaffected by whether headphones or loudspeakers were used. Nevertheless, all participants reported using either in-ear phones or over-the-ear headphones.

### 2.4. Subjective Hearing Assessment

Self-reported (subjective) hearing was assessed by asking participants to rate the question “How would you rate your general hearing abilities?” and the statement “I often experience hearing problems” on an 11-point scale, ranging from “very poor” to “very good” and “strongly disagree” to “strongly agree”, respectively. Whether age groups differed in self-reported hearing was analyzed using Wilcoxon’s rank sum test, and the effect size is provided as rank-biserial correlation [48].

### 2.5. Objective Hearing Assessment: Digits-in-Noise Test

Assessment of pure-tone audiometry can be challenging under remote, including online, testing conditions. Digits-in-noise tests have been proposed as a remote alternative to measuring pure-tone average thresholds [49,50], because digits-in-noise thresholds correlate well with audiometrically measured pure-tone average thresholds (0.5–4 kHz; r > 0.7 [49,51,52,53]). The digits-in-noise test has been used and validated for remote testing, such as by telephone [50,54,55] or smart phone applications [52,56]. Hence, we assessed objective hearing abilities using a digits-in-noise test.

The procedures were mostly similar to those in our previous work [16,57]. Participants listened to digit triplets (e.g., 6-2-4) masked with 12-talker babble noise [58]. Individual digits spoken by a female native English speaker were used to create digit triplets. Digit triplets and babble noise were presented dichotically. The duration of the babble masker was 3 s, and the digit triplet started 0.5 s after babble onset. The digit onset to digit onset was 0.85 s. After the noise and digit triplet ended, participants typed the digits they heard in the order the digits were presented. The signal-to-noise ratio (SNR) was manipulated by varying the level of the spoken digits, whereas the level of the babble noise was the same across trials. Digit triplets were presented at 29 SNRs (range: −18 dB to +15.6 dB; step size: 1.2 dB). One-hundred digit triplets were pre-generated for each of the 29 SNRs by randomly selecting three different digits ranging from 1 to 9. For each participant, twenty-nine digit triplets (one per SNR) were randomly selected. Each participant completed two practice trials with high SNRs (15.6 dB and 14.4 dB SNR), followed by the 29 test trials presented in random order.

For data analysis, a trial was considered correct if all three digits were typed in the order they were presented. A logistic function was fit to the data, and the resulting 50% threshold was used as a dependent measure. An independent samples t-test was used to compare digits-in-noise thresholds between age groups. Previous work provided the regression coefficients that describe the linear relationship between audiometric pure-tone average thresholds (PTAs) and digits-in-noise thresholds (β_1_ = 4.94 and β_0_ = 33.84 for slope and intercept, respectively) [50]. We used Smits et al.’s regression coefficients to provide mean PTAs for both age groups.

### 2.6. AI Voice Experience

The experience with artificial intelligence voice systems was assessed using self-ratings [16]. Participants were asked to rate on a scale ranging from 0 to 10 (no experience and high experience, respectively) the following question: “Do you have experience using a voice-AI (artificial intelligence) system, such as Amazon Alexa, Google Assistant, or Apple Siri”. Age-group differences were assessed using Wilcoxon’s rank sum test, and the effect size is provided as rank-biserial correlation [48].

### 2.7. Data and Statistical Analysis Tools

Data analysis was conducted in the MATLAB software (v2022b), scoring of speech intelligibility was calculated in R (v.2022.12.0) using Autoscore [59], and statistical analyses were conducted in JASP (v0.18.3.0 [60]).

## 3. Experiment 1

Middle-aged to older adults typically have a greater loss of high-frequency (>4 kHz) hearing function than younger adults even when low-frequency hearing thresholds are considered clinically “normal”, that is ≤20–25 dB [24,27,28,61,62]. As a result, high-frequency information in spoken speech may be less accessible to middle-aged to older adults compared to younger adults. Experiment 1 was designed to investigate whether the previously reported age-related decline in identifying AI speech [16] is related to reduced access to high-frequency information in speech for middle-aged to older adults.

### 3.1. Methods

#### 3.1.1. Participants

Fifty-eight younger adults (mean age [std]: 30.6 ± 4.64 years; age range: 22–38 years; 29 male/man and 29 female/woman) and 60 middle-aged to older adults (mean age [std]: 61.3 ± 4.96 years; age range: 54–79 years; 24 male/man and 36 female/woman) participated in Experiment 1. All participants self-identified as native English speakers. Thirty-two younger and 30 middle-aged to older adults listened to unfiltered speech, whereas 26 younger and 30 middle-agedd to older adults listened to low-pass filtered speech, that is, to speech for which frequency information above 4 kHz was suppressed. Sample sizes were determined based on our previous work [16] and a power calculation using GPower with a medium-to-large effect size (α = 0.05, β = 0.95, and f~0.35; estimated N = 109; actual N = 118).

Data quality can be a concern in online studies [63,64,65]. However, with appropriate data quality checks and strict data exclusion criteria (e.g., based on low performance), online research has generally been shown to lead to comparable results as those from studies conducted in-person in a laboratory [65,66,67,68,69,70,71]. In the current study, data from additional participants were recorded but excluded from analysis if they met one or more of the following exclusion criteria: They indicated that they were distracted during the experiment (N = 4), use hearing aids (N = 7), have a history of a neurological disease (N = 4), or reported being a non-native English speaker (N = 1). Participants who passed these exclusion criteria but performed at 0% in the digits-in-noise test (N = 2) had a very high digits-in-noise threshold, i.e., ≥10 (N = 2), or who performed equal to or lower than 80% in the speech-intelligibility task (N = 20) were also excluded, assuming they were unable or unwilling to perform the tasks. Data from one additional participant were also excluded, because the person used the same response category on every trial for their rating of whether a sentence was AI speech or human speech. Overall, 19.7% of the datasets, i.e., 29 out of 147 participants, were excluded. Removal of ~20% of datasets is in line with previous online work that involved comparable screening protocols [16,72].

#### 3.1.2. Speech-Type Categorization Task: Stimuli and Procedures

Eighty sentences from the Harvard sentence lists 1 to 15 were selected. Sentence recordings from 5 female and 5 male human native English speakers were taken from a publicly available resource (https://depts.washington.edu/phonlab/projects/uwnu.php, accessed on 1 January 2024; [73,74]. The speaker IDs for female voices were PNF133, PNF136, PNF140, PNF142, and PNF144, and the speaker IDs for male voices were PNM055, PNM077, PNM078, PNM079, and PNM083 [73,74]. Each sentence was resampled from a sampling frequency of 44.1 kHz to 24 kHz. The Praat software version 6.2.20 [75] was used to calculate each sentence’s duration and median F0.

For Wavenet voices, five female and five male voices with a US English accent (“en-US-Wavenet”) were used. The IDs for female voices were C, E, F, G, and H, whereas the IDs for male voices were A, B, D, I, and J (https://cloud.google.com/text-to-speech/docs/voices, accessed on 1 January 2024). The parameters ‘speaking_rate’ and ‘pitch’ were used to manipulate the duration and median F0, such that for each Wavenet voice, the average duration and median F0 across the sets of 80 sentences did not differ statistically between human and Wavenet speech (for all, *p* > 0.2, with one exception: the median F0 for Wavenet male voice D was larger than the median F0 for PNM078 (*p* < 0.01); the fundamental frequency of PNM078′s voice was very low). In other words, to reduce potential differences between human and Wavenet speech, each Wavenet voice was paired with one human voice of the same gender to adjust Wavenet parameters [16]. Figure 1 depicts an example sentence spoken by a human and computer-generated using Google Wavenet.

Two versions, an unfiltered version and a low-pass filtered version, were created for each sentence. For the low-pass filtered version, sentences were filtered with a low-pass filter that had a pass band below 4 kHz (i.e., hearing loss in older people is most prevalent above 4 kHz [24,77]); a transition band of 4–4.5 kHz; and a cutoff at 4.5 kHz. For the unfiltered version, sentences were not further processed (Figure 1). Participants were randomly assigned to either the ‘unfiltered’ or the ‘filtered’ condition.

For each participant, each of the 80 sentences was randomly assigned to one of the 20 voices (10 human and 10 Wavenet). Hence, throughout the experiment, each participant listened to four sentences for each of the 20 voices. Sentences were presented in four blocks, and each block contained one sentence for each of the 20 voices. On each trial, a fixation cross was presented on the computer screen, while a sentence was presented auditorily in quiet. After each sentence, an input box appeared on the screen, and participants were instructed with the following: “Please type the words exactly as you heard them (even if you only understood parts of what was said)”. This speech-intelligibility task was used to determine whether participants listened to the sentences (and excluded trials or datasets if participants showed low intelligibility). Native English speakers should have no difficulty reporting back verbatim an English sentence in quiet. After typing their response, participants were asked to categorize whether the sentence was spoken by a human or was computer-generated speech (the exact wording was as follows: “Was the voice of the sentence a human or a computer-generated voice?”). Participants could select one out of six response categories presented on the computer screen; the order of the response categories was either as follows or flipped (randomly selected for each participant at the beginning of the experiment): “Human voice (very confident)”, “Human voice (somewhat confident)”, “Human voice (not confident)”, “Computer voice (not confident)”, “Computer voice (somewhat confident)”, and “Computer voice (very confident)”. A 0.4 s blank screen was presented before the next trial started. Replaying sentences was not enabled.

#### 3.1.3. Speech-Type Categorization Task: Data Analysis

Speech intelligibility was analyzed as the proportion of correctly reported words. The scoring of word reports was conducted using the Autoscore package in R [57,59]. The number of correctly reported words was calculated for each sentence and then averaged across sentences, separately for human and Wavenet speech. Data from participants with an overall proportion of correctly reported words lower than 0.8 were excluded from analysis, because all sentences were presented under clear conditions and should thus be highly intelligible when attended to. A repeated-measures analysis of variance (rmANOVA) was calculated using the proportion of correctly reported words as the dependent measure; the within-participants factor Speech Type (human, Wavenet); and the between-participants factors Age Group (younger, middle-aged to older) and Speech Filter (unfiltered, filtered).

The analysis of the performance in the speech-type categorization task focused on sentences for which every word was correctly reported to ensure that only sentences to which participants had listened attentively are included in the analysis (mean and standard deviation of the proportion of trials used for analysis: younger: 0.74 ± 0.14; middle-aged to older: 0.76 ± 0.11). To analyze the proportion of “human voice” responses, the 6-point response and confidence scale was converted, such that “Human voice (very confident)” was coded as 1, “Computer voice (very confident)” as 0, and the other four response categories equally spaced in-between. Responses were averaged across sentences, separately for human and Wavenet speech. An rmANOVA was calculated for the proportion of ‘human voice’ responses using the within-participants factor Speech Type (human, Wavenet) and the between-participants factors Age Group (younger, older) and Speech Filter (unfiltered, filtered).

Perceptual sensitivity (d-prime) was calculated for each participant [78]. A response was considered a hit when the sentence was spoken by a human and the participant responded with “Human voice”. A response was considered a false alarm when the sentence was Wavenet-synthesized and the participant responded with “Human voice”. D-prime cannot be calculated when hit rates or false alarm rates are zero or one [78]. To handle these cases, the following corrections were calculated. When the hit rate was zero, it was set to $\frac{1}{2\times n}$, where n refers to the number of sentences spoken by a human. When the hit rate was one, it was set to $1-\frac{1}{2\times n}$. When the false alarm rate was zero, it was set to $\frac{1}{2\times m}$, where m refers to the number of Wavenet sentences [78]. Data from participants with a false alarm rate of one were removed (N = 1, selecting the same response category on all trials). An rmANOVA was calculated for d-prime using the between-participant factors Age Group (younger, older) and Speech Filter (unfiltered, filtered). D-prime could not be calculated for individual speakers/voices, because the low number of trials per speaker/voice would lead to a frequent false alarm rate of zero, making d-prime less accurate.

### 3.2. Results

#### 3.2.1. Hearing Abilities and Voice-AI Experience

**Figure 2:**
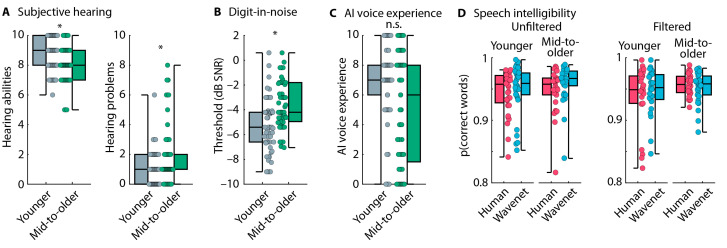
Hearing assessments, voice-AI experience, and speech intelligibility in Experiment 1. (**A**) Subjective (self-rated) hearing abilities (left) and problems (right). (**B**) Digits-in-noise thresholds. (**C**) Self-rated experience with voice-AI systems. (**D**) Proportion of correct word reports in the speech-intelligibility task. The label ‘mid-to-older’ refers to the group of middle-aged to older adults. * *p* ≤ 0.05. n.s.—not significant.

Middle-aged to older adults rated their general hearing abilities as lower (*p* = 0.003, r = 0.309) and their hearing problems to be greater (*p* = 0.019 and r = 0.240; Figure 2A) compared to younger adults. Middle-aged to older adults had worse (i.e., higher) digits-in-noise perception thresholds compared to younger adults (t_116_ = 4.655, *p* = 8.7 × 10^−6^, and d = 0.857; Figure 2B). The digits-in-noise thresholds correspond to an approximate average PTA of 7.8 dB HL for younger adults and a PTA of 16.2 dB HL for middle-aged to older adults [50]. Self-reported experience with voice-AI systems did not differ between the two age groups (*p* = 0.091 and r = 0.179; Figure 2C).

#### 3.2.2. Speech-Type Categorization Task

The proportion of correctly reported words (i.e., speech intelligibility) was greater for Wavenet compared to human speech (effect of Speech Type: F_1,114_ = 4.398, *p* = 0.038, and ω^2^ = 0.004). No other effects or interactions were significant (for all, *p* > 0.05; Figure 2D).

**Figure 3:**
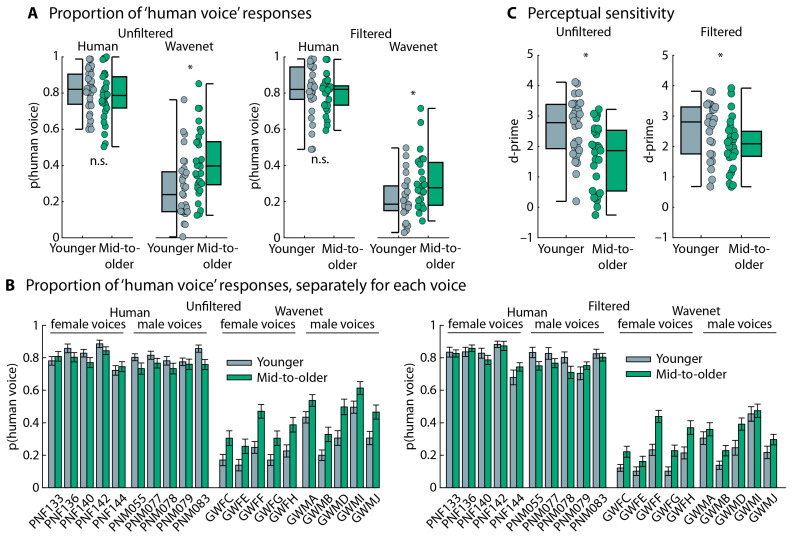
Performance in human vs. AI speech categorization. (**A**) Proportion of ‘human voice’ responses for human and Wavenet voices and for the unfiltered and filtered speech materials. Average responses across the 10 human voices and 10 Wavenet voices. Boxplots and data from each individual (dots) are displayed. (**B**) Proportion of ‘human voice’ responses, separately for the 10 human voices (5 male and 5 female) and the 10 Wavenet voices (5 male and 5 female). Error bars reflect the standard error of the mean. For human voices, abbreviations refer to the labels of original stimulus recordings [73,74]. For Wavenet voices, the first two letters, ‘GW’, of the abbreviation refers to Google Wavenet; the third letter, ‘F’ vs. ‘M’, refers to female and male, respectively; and the fourth letter refers to the specific Wavenet voice (https://cloud.google.com/text-to-speech/docs/voices, accessed on 1 January 2024). (**C**) Perceptual sensitivity across all voices. Boxplots and data from each individual (dots) are displayed. The label ‘mid-to-older’ refers to the group of middle-aged to older adults. * *p* ≤ 0.05. n.s.—not significant.

An analysis of the proportion of “human voice” responses showed that participants responded “human voice” more frequently when the voice was a human voice compared to a Wavenet voice (effect of Speech Type: F_1,114_ = 651.981, *p* = 5.6 × 10^−49^, and ω^2^ = 0.761), as expected. Middle-aged to older adults categorized sentences as originating from a “human voice” more frequently than younger adults (effect of Age Group: F_1,114_ = 7.382, *p* = 0.008, and ω^2^ = 0.027). The Speech Type × Age Group interaction was also significant (F_1,114_ = 12.337, *p* = 6.4 × 10^−4^, and ω^2^ = 0.052). Middle-aged to older adults categorized Wavenet speech more frequently as a “human voice” (t_116_ = 3.763, *p* = 2.7 × 10^−4^, and d = 0.693; Figure 3A) compared to younger adults, whereas there was no difference for human speech (t_116_ = 0.985, *p* = 0.327, and d = 0.181). Participants also categorized filtered speech less frequently as a “human voice” than unfiltered speech (effect of Speech Filter: F_1,114_ = 4.879, *p* = 0.029, and ω^2^ = 0.017), although this was only significant for Wavenet speech (t_116_ = 2.448, *p* = 0.016, and d = 0.451) and not for speech spoken by a human (t_116_ = 0.181, *p* = 0.857, and d = 0.033; Speech Type × Speech Filter interaction: F_1,114_ = 4.870, *p* = 0.029, and ω^2^ = 0.019). None of the other interactions were significant (for all, *p* > 0.4). Figure 3B depicts the proportion of “human voice” responses separately for each voice and speech condition. The plots indicate that the difference between younger and middle-aged to older adults generalizes across voices.

Perceptual sensitivity (d-prime) was calculated as a bias-free measure of a person’s ability to categorize human vs. AI speech. Younger adults were more sensitive to human vs. AI speech compared to middle-aged to older adults, as indicated by larger d-prime values (F_1,114_ = 17.435, *p* = 5.8 × 10^−5^, and ω^2^ = 0.121; Figure 3C). The Speech Filter effect (F_1,114_ = 1.032, *p* = 0.312, and ω^2^ < 0.001) and the Speech Filter × Age Group interaction were not significant (F_1,114_ = 1.649, *p* = 0.202, and ω^2^ = 0.005), suggesting that d-prime was unaffected by the suppression of high-frequency information in the speech materials.

#### 3.2.3. Summary

Experiment 1 replicates previous work that shows middle-aged to older adults are less able to distinguish between human and AI speech [16]. Critically, middle-aged to older adults identified AI speech less well than younger adults for both filtered and unfiltered speech. The results indicate that any high-frequency hearing loss, which is typically present in middle-aged to older but not in younger adults [24,27,61,62], may not contribute to the reduced ability of older adults to identify AI speech.

## 4. Experiment 1 Follow-Up

In Experiment 1, after the presentation of a sentence, participants performed the speech-intelligibility task before the speech-type categorization task. After the study was concluded, a potential concern was considered, namely, that middle-aged to older adults may have performed more poorly in categorizing AI speech than younger adults because of lower task-switching or memory abilities [79,80,81]. To address this potential concern, we conducted a follow-up experiment in which the task order was reversed, such that participants first performed the speech-type categorization task and then the speech-intelligibility task.

### 4.1. Methods

#### 4.1.1. Participants

Twenty-six younger adults (mean age [std]: 31.4 ± 4 years; range: 21–36 years; 10 male/man, 14 female/woman, 1 non-binary, and 1 trans feminine) and 27 middle-aged to older adults (mean age [std]: 59.9 ± 6.5 years; range: 53–74 years; 12 male/man, 14 female/woman, and 1 non-binary) participated in the follow-up experiment (11 additional datasets were recorded but excluded from the analysis based on the same criteria used in Experiment 1).

#### 4.1.2. Procedures

Participants ran through the same experimental procedures as for Experiment 1. The critical difference during the main task procedures was that after each sentence, participants first categorized whether the sentence was spoken by a human or was computer-generated speech and subsequently provided verbatim word reports (speech intelligibility). Only the unfiltered speech condition was used in this follow-up experiment.

### 4.2. Results

**Figure 4:**
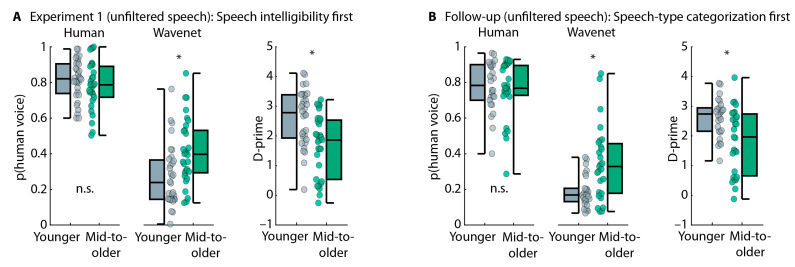
Results for follow-up experiment and comparison to results from Experiment 1. (**A**) Proportion of ‘human voice’ responses for human and Wavenet speech and perceptual sensitivity (d-prime) for data from Experiment 1. After each sentence, participants provided verbatim word reports (intelligibility), followed by the speech-type categorization (human vs. computer-generated). (**B**) Same as for panel (**A**). In the follow-up experiment, after each sentence, participants categorized the speech first (human vs. computer-generated) and then provided verbatim word reports (intelligibility). The results are very similar. The label ‘mid-to-older’ refers to the group of middle-aged to older adults. * *p* < 0.05. n.s.—not significant.

Middle-aged to older adults categorized Wavenet speech more frequently as human speech than younger adults (t_51_ = 3.655, *p* = 6.1 × 10^−4^, and d = 1.004), whereas no age-group difference was found for human speech (t_51_ = 0.416, *p* = 0.679, and d = 0.114; Speech Type × Age Group interaction: F_1,51_ = 8.583, *p* = 0.005, and ω^2^ = 0.068). Perceptual sensitivity (d-prime) was higher in younger adults compared to middle-aged to older adults (t_51_ = 3.060, *p* = 0.004, and d = 0.841; Figure 4B). These results thus replicate those from Experiment 1.

To analyze the potential task order effect on d-prime, we combined data from Experiment 1 and the follow-up experiment. The analysis revealed no significant differences in d-prime between Experiment 1 and the follow-up experiment (effect of Experiment: F_1,111_ = 0.178, *p* = 0.674, and ω^2^ < 0.001; Experiment × Age Group interaction: F_1,111_ = 0.225, *p* = 0.638, andω^2^ < 0.001; Figure 4), whereas the Age Group effect was significant (F_1,111_ = 22.672, *p* = 5.8 × 10^−6^, and ω^2^ < 0.001). Hence, the data provide no evidence that the order of the tasks affects the current results.

## 5. Experiment 2

Previous work suggests that older adults are less able to use prosodic information in speech, for example, to make judgements about the emotions conveyed through speech [30,31,34,36,37,39]. Experiment 2 investigates whether this reduction in the processing of prosodic information in speech predicts the age-related reduction in AI speech identification. This would provide evidence for an important role of processing prosodic information for the identification of AI speech.

### 5.1. Methods

#### 5.1.1. Participants

Seventy-five younger adults (mean [std]: 31.8 ± 3.38 years; range: 23–37 years; 40 female/woman, 30 male/man, 2 non-binary, and 1 no information) and 86 middle-aged to older adults (mean [std]: 61.5 ± 5.42 years; range: 55–79 years; 65 female/woman, 20 male/man, and 1 genderqueer) participated in Experiment 2. All participants identified as native English speakers. Analyses of Experiment 2 involved a regression to predict AI speech-categorization performance from the emotion-categorization performance. Sample sizes were based on power calculations using GPower for a linear multiple regression with 4 predictors (including other relevant variables), assuming a medium-to-large effect size (α = 0.05, β = 0.95, and f^2^~0.25; estimated N = 80; actual N = 161; estimated N = 73 per group for group-separate regressions with 3 predictors (not including age groups)).

Additional participants’ data were excluded if they indicated that they were distracted (N = 2), use hearing aids (N = 5), have a history of a neurological disease (N = 3), or are a non-native English speaker (N = 1). Participants who had 0% performance in the digits-in-noise test (N = 3), a digits-in-noise threshold of ≥10 (N = 5), performed equal to or lower than 80% in the speech-intelligibility part of the AI speech categorization task (N = 21), or scored equal to or lower than 60% in word intelligibility in the emotion-categorization task (N = 18) were also excluded, assuming they were unable or unwilling to perform the tasks. Note that the 60% threshold for intelligibility in the emotion-categorization task was chosen, because participants scored lower overall for reporting the one word (cf. [82]) compared to the several words of a sentence in the AI speech categorization task. Overall, 18.3% of the datasets, i.e., 36 out of 197 participants, were excluded from further analysis.

#### 5.1.2. Speech-Type Categorization Task

The stimuli and procedures were the same as in Experiment 1, with the exception that human-spoken speech from six instead of ten speakers was used in Experiment 2 (3 female: PNF136, PNF142, and PNF144; 3 male: PNM055, PNM79, and PNM83 [73,74]). The six Google Wavenet voice IDs in Experiment 2 were F, G, and H for female voices and A, B, and J for male voices. We reduced the number of voices from 10 (Experiment 1) to 6 (Experiment 2) to partially offset the additional time needed by participants to complete the emotion-categorization task. The 6 specific speakers and 6 voices were selected, because sound features (i.e., duration and fundamental frequency) matched well across human and Wavenet speech. In Experiment 2, each participant listened to 48 sentences overall. Each of the 48 sentences was randomly assigned to one of the 12 voices (6 human and 6 Wavenet). Sentences were presented in two blocks, and each block contained two sentences for each of the 12 voices.

Speech intelligibility, the proportion of “human voice” responses, and perceptual sensitivity were calculated as in Experiment 1, using only sentences for which every word was correctly reported (mean and standard deviation of the proportion of trials used for analysis: younger: 0.81 ± 0.14; middle-aged to older: 0.79 ± 0.11).

#### 5.1.3. Emotion-Categorization Task

To examine the ability of individuals to identify emotions conveyed through prosodic information in speech, we used the Toronto Emotion Speech Set (TESS [42,82,83]). TESS encompasses stimuli spoken by two female voice actors, an older adult aged 60 and a younger adult aged 20 (native English speakers). Both voice actors uttered seven different emotions through prosody (Angry, Disgust, Fear, Happy, Neutral, Pleasantly Surprised, and Sad) for a collection of 200 emotionally neutral words (e.g., back, pass, came, etc. [84,85]; 200 words × 2 actors × 7 emotions). The words were supplemented by a standard carrier sentence “Say the word…” preceding the target word (e.g., “Say the word back”). In the present study, we selected 168 stimuli (24 words × 1 actor × 7 emotions) from the TESS. The 24 words used in the current study were selected from the 200 words of the full TESS dataset by removing items that had high valence ratings and those for which younger and older adults showed low performance [42,82,83] and by restricting target words to four-letter words. We opted for the recordings of the younger voice actor, because previous research demonstrated smaller age-related differences in emotion categorization for the speech from the younger voice actor compared to the older voice actor [83].

Participants performed two blocks in the emotion-categorization task, including 84 trials each. For each trial, participants listened to a sentence from the speech emotion test set (e.g., “Say the word… PASS”). After each sentence, their keyboard was used to type the final (target) word (i.e., PASS in the example). The word report was used to assess speech intelligibility in the emotion-categorization task. After they typed the word, participants were asked to categorize the emotion conveyed (“Which emotion was conveyed?”). Participants were presented with the following response options: anger, disgust, fear, happiness, neutrality, pleasant surprise, and sadness. In each of the two blocks, 12 trials for each of the seven emotion types were presented. The order in which stimuli were presented was randomized for each participant.

Speech intelligibility for target words was assessed as the proportion of correctly reported words. An independent t-test was calculated to compare age groups. The performance in the emotion-categorization task was assessed as the proportion of correctly identified emotions, using only trials for which the word was correctly reported in the speech-intelligibility task (mean and standard deviation of the proportion of trials used for analysis: younger: 0.89 ± 0.05; middle-aged to older: 0.84 ± 0.09). An independent t-test was calculated to compare age groups.

#### 5.1.4. Relationship Between Performance in Speech-Type and Emotion Categorization Tasks

The relationship between the performance in the speech-type categorization task and the performance in the emotion-categorization task was investigated using a linear regression analysis to account for possible influences of age group, hearing abilities (digits-in-noise thresholds), and experience with voice-AI systems. D-prime from the speech-type categorization task (AI vs. human speech) was used as the dependent variable in the regression analysis. Predictors were the proportion of correctly identified emotions in the emotion-categorization task, age group, digits-in-noise threshold, and self-rated experience with voice-AI systems. Linear regression analyses were also calculated separately for younger and middle-aged to older adults to investigate whether the hypothesized relationship between speech-type categorization performance and emotion-categorization performance exists for one or both age groups.

### 5.2. Results

#### 5.2.1. Hearing Abilities and Voice-AI Experience

**Figure 5:**
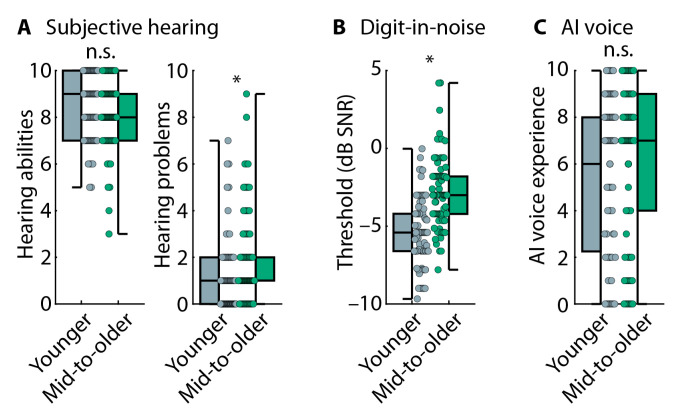
Hearing assessments and voice-AI experience. (**A**) Subjective (self-rated) hearing abilities (left) and problems (right). (**B**) Digits-in-noise threshold. (**C**) Self-rated experience with voice-AI systems. In Panels (**A**–**C**), boxplots and data from each individual (dots) are displayed. The label ‘mid-to-older’ refers to the group of middle-aged to older adults. * *p* ≤ 0.05. n.s.—not significant.

Middle-aged to older adults rated having more hearing problems compared to younger adults (*p* = 0.004 and r = 0.256), but age groups did not differ for self-rated general hearing abilities (*p* = 0.076 and r = 0.266; Figure 5A). Middle-aged to older adults also had higher (i.e., worse) digits-in-noise perception thresholds compared to younger adults (t_159_ = 7.642, *p* = 1.9 × 10^−12^, and d = 1.207; Figure 5B). The digits-in-noise perception thresholds correspond to an approximate average PTA of 7 dB HL for younger adults and a PTA of 20.1 dB HL for middle-aged to older adults [50]. There was no difference in self-rated voice-AI experience between age groups (*p* = 0.248 and r = 0.105; Figure 5C).

#### 5.2.2. Speech-Categorization Task

**Figure 6:**
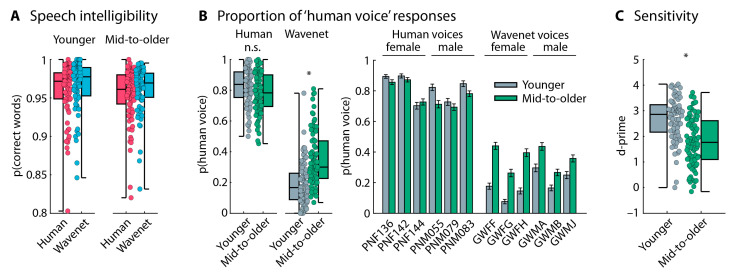
Performance in speech intelligibility and human vs. AI speech categorization. (**A**) Proportion of correctly reported words in the speech-intelligibility task. (**B**) (left) Proportion of ‘human voice’ responses for human and Wavenet voices. Average responses across the 6 human voices and 6 Wavenet voices. (Right) Proportion of ‘human voice’ responses, separately for the 6 human voices (3 male and 3 female) and the 6 Wavenet voices (3 male and 3 female). Error bars reflect the standard error of the mean. For human voices, abbreviations refer to the labels of original stimulus recordings [73,74]. For Wavenet voices, the first two letters, ‘GW’, of the abbreviation refers to Google Wavenet; the third letter, ‘F’ vs. ‘M’, refers to female and male, respectively; and the fourth letter refers to the specific Wavenet voice (https://cloud.google.com/text-to-speech/docs/voices, accessed on 1 January 2024). (**C**) Perceptual sensitivity across voices. Boxplots and data from each individual (dots) are displayed. The label ‘mid-to-older’ refers to the group of middle-aged to older adults. * *p* ≤ 0.05. n.s.—not significant.

Speech intelligibility for the sentences was higher for Wavenet than human speech (effect of Speech Type: F_1,159_ = 10.595, *p* = 0.001, and ω^2^ = 0.011) but did not differ between age groups (effect of Age Group: F_1,159_ = 0.972, *p* = 0.326, and ω^2^ < 0.001). There was no Speech Type × Age Group interaction (F_1,159_ = 0.231, *p* = 0.631, and ω^2^ < 0.001; Figure 6A).

Data from the speech-categorization task (human vs. computer voice) are displayed in Figure 6B. Participants categorized speech more frequently as human speech when it was spoken by a human compared to being computer generated (F_1,159_ = 1059.649, *p* = 3.3 × 10^−72^, and ω^2^ = 0.774), as expected. Middle-aged to older adults categorized speech more frequently as human speech than younger adults (F_1,159_ = 15.481, *p* = 1.2 × 10^−4^, and ω^2^ = 0.043). The Speech Type × Age Group interaction was also significant (F_1,159_ = 38.141, *p* = 5.3 × 10^−9^, and ω^2^ = 0.107), because middle-aged to older adults categorized Wavenet speech more frequently as human speech than younger adults (t_159_ = 6.434, *p* = 1.4 × 10^−9^, and d = 1.017) and human speech less frequently as human speech, although this was only marginally significant (t_159_ = 1.962, *p* = 0.051, and d = 0.310). Perceptual sensitivity (d-prime) was higher in younger adults compared to middle-aged to older adults (t_159_ = 6.336, *p* = 2.3 × 10^−9^, and d = 1; Figure 6C). These results replicate the results from Experiment 1 and previous work [16].

#### 5.2.3. Emotion-Categorization Task

**Figure 7:**
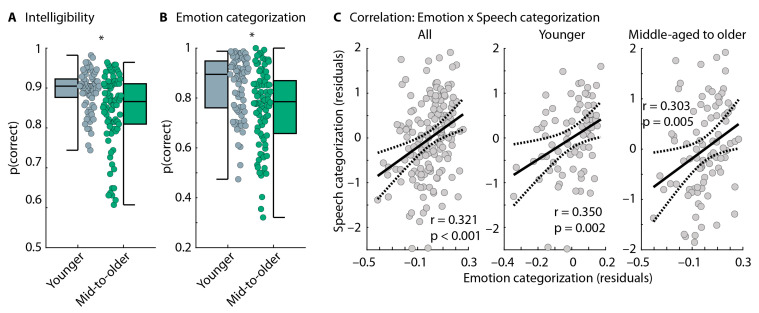
Results from the emotion-categorization task and correlation with speech-categorization performance. (**A**) Intelligibility for target words in the emotion-categorization task. (**B**) Performance in the emotion-categorization task. (**C**) Correlation between emotion-categorization performance and speech-categorization performance (human vs. computer voice), including all participants (**left**), younger adults (**middle**), or older adults (**right**). Data reflect the residuals after regressing out age group (**left**), digits-in-noise threshold, and voice-AI experience. The solid line reflects the best linear fit and the dashed lines the confidence interval. The label ‘mid-to-older’ refers to the group of middle-aged to older adults. * *p* ≤ 0.05.

Intelligibility for target words in the emotion-categorization task was lower for middle-aged to older adults compared to younger adults (t_159_ = 4.295, *p* = 3 × 10^−5^, and d = 0.679: Figure 7A). For the emotion-categorization task, we analyzed only words that were correctly reported in the intelligibility task. Middle-aged to older adults categorized emotions conveyed through speech prosody less well than younger adults (t_159_ = 3.869, *p* = 1.6 × 10^−4^, and d = 0.611; Figure 7B), consistent with the original studies from which the stimulus materials were derived [42]. The age-related decline was present for positive, neutral, and negative emotions, although this was significant only for positive (t_159_ = 2.817, *p* = 0.006, and d = 0.445) and negative emotions (t_159_ = 4.150, *p* = 5.4 × 10^−5^, and d = 0.656) but not the neutral emotion (t_159_ = 1.169, *p* = 0.244, and d = 0.185), mainly because performance was closer to the ceiling for neutral stimuli (effect of Emotion: F_2,318_ = 20.413, *p* = 4.6 × 10^−9^, and ω^2^ = 0.046; the Emotion × Ag Group interaction as not significant: F_2,318_ = 2.096, *p* = 0.125, and ω^2^ = 0.003).

#### 5.2.4. Correlation Between Speech-Categorization Performance and Emotion-Categorization Performance

Experiment 2 aimed to investigate the relationship between the ability of individuals to categorize speech (human vs. computer voice) and their ability to categorize emotions conveyed through speech prosody. The linear regression model including younger and middle-aged to older adults was significant (R^2^ = 0.328, F_4,156_ = 19.075, and *p* = 8.7 × 10^−13^). Better performance in the emotion-categorization task significantly predicted better performance in the speech-type categorization task (t_156_ = 4.231 and *p* = 4 × 10^−5^; Figure 7C). Age group also predicted speech-type categorization performance (t_156_ = −3.509 and *p* = 5.9 × 10^−4^): performance was lower for middle-aged to older than younger adults, as expected, based on the analyses reported above (Figure 6C). The digits-in-noise threshold showed a small effect as well (t_156_ = −2.168 and *p* = 0.032). Self-rated experience with voice-AI systems did not predict the performance in speech-type categorization (t_52_ = −0.38 and *p* = 0.706). The linear regression models for both groups, separately, were also significant: younger adults (R^2^ = 0.176, F_3,71_ = 5.050, and *p* = 0.003) or middle-aged to older adults (R^2^ = 0.150, F_3,82_ = 4.838, and *p* = 0.004). The effect of emotion-categorization performance on speech-type categorization performance was significant (younger: t_71_ = 3.144 and *p* = 0.002; middle-aged to older: t_82_ = 2.880 and *p* = 0.005; Figure 7C), whereas effects of digits-in-noise threshold and experience with voice-AI systems were not significant (*p* > 0.05).

These results were confirmed by an explorative mediation analysis in which we examined the extent to which emotion identification (emotion-categorization performance), hearing abilities (digits-in-noise threshold), and experience with voice-AI systems mediated the relationship between biological age and AI speech identification (d-prime in the speech-type categorization task). Emotion identification mediated the relationship (*p* = 0.002), whereas hearing abilities and voice-AI experience showed no mediating effect (for both, *p* > 0.05). However, biological age still had a direct effect on AI speech identification (*p* = 5.7 × 10^−5^), suggesting that additional factors associated with aging contribute to the decline in AI speech identification.

#### 5.2.5. Summary

Experiment 2 replicated previous observations that middle-aged to older adults identify AI speech (Experiment 1) [16] and emotions from speech prosody [42] less well than younger adults. Critically, the current results show that individuals who better identify emotions from prosodic information in speech are also better at identifying AI speech, suggesting a critical role of prosodic processing in AI speech identification, independent of hearing abilities and experience with voice-AI systems.

## 6. General Discussion

Previous research demonstrated that middle-aged to older adults are less able to identify modern AI speech compared to younger adults [16]. The current study investigated potential factors that could explain this age-related reduction in AI speech identification.

### 6.1. Older Adults Are Less Able to Identify Modern AI-Based Synthesized Speech

The current study replicates previous work [16], showing that middle-aged to older adults are less able to identify whether a sentence they heard was spoken by a human or was computer-generated using a modern AI-based speech synthesizer (Figure 3, Figure 4 and Figure 6). Critically, this analysis focused only on the sentences for which all words were correctly reported, thus excluding group differences in intelligibility as a contributor. In our follow-up experiment, participants judged immediately after each sentence whether the speech was spoken by a human or was computer-generated. The results replicated the age-related reduction in AI speech identification (Figure 4) and thus make contributions from potential age-group differences in task-switching or phonological or other memory abilities unlikely [79,80,81]. Moreover, we did not observe age-group differences in self-rated experience with voice-AI systems, similar to previous work [15,16], and self-rated experience did not predict speech-categorization performance. This suggests that experience with voice-AI systems does not contribute to the age-related reduction in speech-categorization performance. This interpretation is also consistent with work showing that familiarizing individuals with synthesized speech only minimally improves the detectability of synthesized speech [86].

In the current study, we used Google’s Wavenet voices [5], which sound very naturalistic and are far beyond the robotic-sounding voices that were common a few years ago (for naturalness ratings for these voices, see [16]). Nevertheless, voice-AI technology continues to develop, and since the data for the current study were recorded, several updated voice versions have been introduced (https://cloud.google.com/text-to-speech/docs/voices). Several big-tech companies (Google, OpenAI, Meta, Amazon, and Microsoft) have introduced AI-based text-to-speech synthesizers that generate remarkably naturalistic speech, highlighting the wide interest in this technology. This technology is fast advancing, such that synthesized speech is becoming increasingly hard to recognize as such. Identifying whether speech is spoken by a human or is computer-generated will likely become more difficult, not just for middle-aged and older adults, as shown in the current study, but also for younger adults. In the future, AI speech identification will likely require taking into account information from both voice properties as well as speech content.

### 6.2. Hearing Loss Is Unlikely to Explain the Age-Related Reduction in AI Speech Identification

The results of Experiment 1 show that middle-aged to older adults were less able to identify AI speech than younger adults for both full-bandwidth speech and 4 kHz low-pass filtered speech. In other words, sensitivity to AI speech appears to be unaffected by the removal of high-frequency information (Figure 3C), to which older adults are typically less sensitive due to hearing loss [24,27,61,62]. These results provide little support for the idea that high-frequency hearing loss (>4 kHz) in middle-aged to older adults contributes to the reduced ability to identify AI speech in this group. This is further corroborated by the small effect of Speech Filter in the analysis of the proportion of “human voice” responses (Figure 3A), which indicated that participants were more likely to perceive the Wavenet voice as computer-generated (AI) speech when high-frequency information was removed, suggesting that high-frequency speech information interferes rather than helps with AI speech identification.

It should be noted that the experimental procedures in the current study were conducted online, and pure-tone audiometry at different frequencies could not be administered this way. Instead, we estimated participants’ pure-tone average thresholds (PTA: 0.5–4 kHz) by measuring digits-in-noise perception thresholds and converting them to the PTA [50]. Digits-in-noise thresholds have been shown to correlate highly with the PTA [50,54,55]. The mean estimated PTA for younger adults was ~7 dB HL and ~18 dB HL for middle-aged to older adults. These estimates are consistent with previous in-lab work of community-dwelling middle-aged to older adults and are typical for middle-aged to older adults who also have some degree of high-frequency hearing loss [61,87,88,89,90]. Moreover, epidemiological studies indicate that most middle-aged to older people in the current age range will have some degree of high-frequency hearing loss [17,18,19,29]. Regardless of the extent of high-frequency hearing loss in the middle-aged to older adults who participated in the current study, it appears that high-frequency information in speech contributes little to AI speech identification, and if so, then it appears to provide interfering information.

Finally, that the digits-in-noise thresholds in Experiment 2 did not predict AI speech-identification (see also [16]), or only very little (i.e., when participants from both age groups were included in a pooled analysis), further corroborates the conclusion that hearing loss is probably not the main driver for reduced AI speech identification in middle-aged to older adults.

### 6.3. Age-Related Decline in Prosody Processing Is Associated with Reduced AI Speech Identification

In Experiment 2, we showed that words spoken with emotional prosodic intonation are less intelligible for middle-aged to older adults than younger adults and that middle-aged to older adults are less able to identify different emotions conveyed through prosodic speech information (Figure 6A,B). Both observations replicate previous work from which the stimulus materials were taken [42,82,83], with the broader literature reporting age-related changes in the processing of emotional prosody in speech [30,31,32,33,34,35,36,37,38,39,40,41,91,92,93]. Critically, the current analysis of emotion categorization was conducted for correctly heard words, suggesting that emotion identification is reduced in older adults even for intelligible speech.

Recent meta-analytic work suggests a positivity bias, such that older adults experience difficulties especially in recognizing negative emotions [94]. Here, we observed no difference in the age-related decline for positive and negative emotions, although numerically (i.e., non-significantly), the mean difference was greater for negative emotions. Studies included in the meta-analysis varied widely in terms of stimulus materials and sensory modality [94], potentially explaining that we did not find a positivity bias here. Future work may need to further elaborate the age-related positivity bias for emotion recognition based on prosodic speech information.

Experiment 2 further demonstrated that listeners who were less able to identify different emotions from the prosodic information in speech were also less able to identify AI speech (Figure 7). The data show that this relationship is not limited to older adults but is present also for younger people. Hence, even younger adults who perform more poorly at identifying emotions from speech prosody identify AI speech less well. Since processes related to aging reduce the ability to identify emotions in speech, it might then not be surprising—given the observed relationship between prosody processing and AI speech identification—that middle-aged to older adults are also more likely to have difficulties with the identification of AI speech. That younger adults—who typically have normal hearing—show the relationship between prosodic processing and AI speech identification further highlights that hearing loss may not be the main driver of the observed age-related reduction in AI speech identification.

The relationship between processing prosodic information in speech and AI speech identification, however, does not provide a clear mechanistic explanation of the underlying causes. Some works suggest that a reduced ability to process emotional prosody is related to sensory impairments [30], whereas other work points to non-sensory causes [42,43,44,45]. Moreover, the current study cannot distinguish between emotional processing and prosodic processing per se, because the TESS stimuli and task used here assess emotion-identification through prosody. Future studies may aim to separate these two factors and investigate the relative contributions of the processing of emotions (e.g., conveyed through speech semantics or facial expressions) and prosody (e.g., discriminations of the fundamental frequency of speech). In addition, a wide range of acoustic features may support recognizing emotions from prosodic cues [95], and future research might examine the importance of specific prosodic and acoustic cues for AI speech recognition. Nevertheless, the current data suggest that the mechanisms underlying the processing of emotional prosody in speech may also, at least partially, underlie the identification of AI speech, thereby providing directions for future investigations.

## 7. Conclusions

The current study investigated why middle-aged to older adults, relative to younger adults, are less able to identify modern AI-based synthesized speech. In two experiments, we showed that experience with voice-AI systems and age-related hearing loss are unlikely explanations of the age-related reduction in AI speech identification. Instead, we showed that a lower ability to capitalize on prosodic information in speech—which is more prevalent in middle-aged to older adults—predicts poorer AI speech identification. The results of the current study suggest that the accurate processing of prosodic information in speech facilitates the identification of modern AI-based synthesized speech.

## Data Availability

The data are available in the Open Science Framework repository: https://osf.io/n6svd/, accessed on 1 January 2024.
